# Construction of a Novel Oxidative Stress Response-Related Gene Signature for Predicting the Prognosis and Therapeutic Responses in Hepatocellular Carcinoma

**DOI:** 10.1155/2022/6201987

**Published:** 2022-09-12

**Authors:** Junjie Hong, Xiujun Cai

**Affiliations:** Key Laboratory of Laparoscopic Technique Research of Zhejiang Province, Department of General Surgery, Sir Run Run Shaw Hospital, Zhejiang University School of Medicine, Hangzhou 310016, China

## Abstract

Hepatocellular carcinoma (HCC) is a highly heterogeneous malignancy with poor outcomes, and the assessment of its prognosis as well as its response to therapy is still challenging. In this study, we aimed to construct an oxidative stress response–related genes–(OSRGs–) based gene signature for predicting prognosis and estimating treatment response in patients with HCC. We integrated the transcriptomic data and clinicopathological information of HCC patients from The Cancer Genome Atlas (TCGA) and the International Cancer Genome Consortium (ICGC) databases. LASSO Cox regression analysis was utilized to establish an integrated multigene signature in the TCGA cohort, and its prediction performance was validated in the ICGC cohort. The CIBERSORT algorithm was employed to evaluate immune cell infiltration. The response rate to immune checkpoint inhibition (ICI) therapy was assessed using a TIDE platform. Drug activity data from the Cancer Genome Project and NCI-60 human cancer cell lines were used to predict sensitivity to chemotherapy. We successfully established a gene signature comprising *G6PD*, *MT3*, *CBX2*, *CDKN2B*, *CCNA2*, *MAPT*, *EZH2*, and *SLC7A11*. The risk score of each patient, which was determined by the multigene signature, was identified as an independent prognostic marker. The immune cell infiltration patterns, response rates to ICI therapy, and the estimated sensitivity of 89 chemotherapeutic drugs were associated with risk scores. Individual prognostic genes were also associated with susceptibility to various FDA-approved drugs. Our study indicates that a comprehensive transcriptomic analysis of OSRGs can provide a reliable molecular model to predict prognosis and therapeutic response in patients with HCC.

## 1. Introduction

Hepatocellular carcinoma (HCC) ranks fifth among all malignancies worldwide and is the second most common cause of cancer-associated deaths [1]. Despite advances in therapeutic measures, the prognosis of HCC has improved very little over the last two decades, and owing to the absence of specific signs and clinical symptoms, most often, HCC is diagnosed when it is already at advanced stages, implying that many patients miss out on the opportunity to receive surgery, which is more effective as a therapeutic measure at the earlier stages of the disease. However, for patients with advanced HCC, comprehensive treatment, including chemotherapy, targeted therapy, and immunotherapy, are crucial for improving prognosis, and in these cases, physicians have limited tools that can help guide the treatment decision-making process. Specifically, in actual clinical contexts, prognosis prediction and treatment recommendation highly depend on patients' clinical characteristics and pathological features. For instance, the Barcelona clinic liver cancer (BCLC) staging system, which is based on the Child-Pugh score, tumor size, and performance status, is one of the most used staging algorithms for HCC with prognostic and therapeutic significance. However, its rigidity limits its accuracy and effectiveness due to the high interpatient heterogeneity of HCC [2]. Hence, novel prognostic markers that can better inform treatment strategies and adequately predict the prognosis are urgently needed. The recent technological progress in microarray and RNA sequencing has allowed the integration of multiple prognosis-related genes into a single prognostic model, providing a powerful approach to improve prediction accuracy in prognosis and treatment response.

It is well known that cancer cells are generally under stressful biological conditions, among which oxidative stress is one the most representative [3]. Increased oxidative stress is a hallmark of the cancer microenvironment, reflected by the elevated intracellular levels of reactive oxygen species (ROS) [4, 5]. ROS are highly reactive oxygen chemicals comprising peroxides, superoxide, hydroxyl radicals, and hydrogen peroxide. Dysregulated ROS in cancer cells may be attributed to the activation of the oncogene, hypoxia, and extracellular stimuli, such as chemotherapy and radiotherapy [6–8]. Excessive generation of ROS can be lethal to cancer cells, as it induces cell apoptotic death via DNA repair disorders, protein damage, and lipid peroxidation [9]. From the early stage of carcinogenesis, cancer cells utilize endogenous adaption to survive oxidative stress, including transcriptomic and proteomic modulation [10]. Earlier studies reported that multiple genes, known as oxidative stress response-related genes (OSRGs), are involved in the above biological process and are essential for the proliferation, progression, and migration of HCC cells [5]. For instance, the proteins encoded by *MGST1/3*, *G6PD*, *GSR*, and *GPX2/4* play vital roles in glutathione synthesis, which serves as an antioxidant defender in cancer cells [11]. Besides, TXN, together with PRDXs, reduces intracellular hydrogen peroxide and oxidized proteins [12]. Generally, these genes are upregulated in tumor tissues and indicate a poor prognosis, making them promising therapeutic targets.

Even though OSRGs are essential for the survival of HCC cells, the prognosis prediction and therapy recommendation value of OSRGs have not yet been sufficiently elucidated. Therefore, in this study, our aim was to investigate the prognosis prediction and therapy recommendation value of OSRGs. Thus, we successfully developed a novel eight OSRGs-based gene signature for HCC. First, we integrated transcriptomic profiling data and clinicopathological information related to HCC from The Cancer Genome Atlas (TCGA) and the International Cancer Genome Consortium (ICGC) databases. Next, we filtered the OSRGs related to overall survival (OS) in the TCGA cohort and established an eight-gene signature, whose robustness was then validated using the ICGC cohort. We also analyzed the correlation between tumor-infiltrating immune cells and risk scores. Further, immune checkpoint expression, immune checkpoint inhibition (ICI) therapy response rate, and tumor mutation burden (TMB) scores were evaluated to identify potentially valid immunotherapies. Finally, chemotherapeutic susceptibility was estimated according to the risk scores obtained and the expression levels of the individual prognostic genes. In summary, our work not only developed a practical prognostic tool but also provided a novel and reliable gene signature for selecting appropriate therapeutic methods for HCC. These innovative discoveries offer promising prospects for enhancing antitumor effects as each patient can receive individualized treatment guidance through our developed gene signature.

## 2. Materials and Methods

### 2.1. Databases

We downloaded the transcriptomic and clinical data of HCC from the Liver Hepatocellular Carcinoma (TCGA-LIHC) dataset in the TCGA database and the LIRI-JP cohort in the ICGC database. Both TCGA and ICGC databases are publicly available; hence, an ethical review is not required for their use. OSRGs were retrieved from the MSigDB database v7.4 (https://www.gsea-msigdb.org/gsea/msigdb/) (Table S1). The expression intensity and distribution of specific proteins in HCC and normal liver tissue were evaluated using clinical specimens from the Human Protein Atlas (HPA) database (https://www.proteinatlas.org/).

### 2.2. Construction and Validation of the Prognostic Oxidative Stress Response Genes Signature

We identified the differentially expressed genes (DEGs) between the normal liver tissue and HCC tissues in the TCGA cohort using the “limma” package in R software Version 4.0.2 with the following threshold values: |log2FC| ≥ 1 and adj.p < 0.05. The shared candidate genes of DEGs and OSRGs were analyzed using univariate Cox regression analysis, genes with adj.p < 0.001 were preserved. Then, LASSO Cox regression analysis was adopted to construct a prognostic gene signature, using R software with “glmnet” package. The risk score of each patient was determined as follows: Risk score = ∑(Coef(i)∗Expression of gene(i)). The Kaplan-Meier (K-M) curve was plotted using the “survminer” and “survival” packages in R software. We delineated the receiver operating characteristic (ROC) curves with the “timeROC” package in R software to assess the accuracy of the prognostic gene signature.

### 2.3. Nomogram Establishment

A forecast nomogram was developed by incorporating risk scores, age, gender, stage, and TNM classification using the R package “RMS.” The one-, two-, and three-year OS probabilities were estimated using the total points. Calibration curves were used to evaluate the accuracy.

### 2.4. Assessment of Immune Cell Infiltration

We used the CIBERSORT algorithm to quantify the subsets of infiltrating immune cells in the tumor environment of HCC samples. CIBERSORT is a deconvolution algorithm that uses the gene expression matrix to calculate the proportion of specific types of immune cells [13].

### 2.5. Prediction of Immune Checkpoint Inhibition Therapy

The tumor immune dysfunction exclusion (TIDE) analysis platform was utilized to generate the TIDE score of each patient, which could serve as a surrogate biomarker to predict the responses to ICI therapy, mainly including anti-PD1 and anti-CTLA4 therapy (http://tide.dfci.harvard.edu/) [14]. A high TIDE score indicates a low response rate to ICI therapy.

### 2.6. Calculation of TMB Scores

TMB is a measure of gene mutation frequency in a cancer cell. We obtained the VarScan processed somatic mutation data of HCC from TCGA database. Strawberry-Perl version 5.30.1 based on the JAVA8 platform was used to determine the TMB score depending on the genome mutation information of each HCC sample.

### 2.7. Chemotherapy Response Estimation

The R package “pRRophetic” was adopted to calculate the half-maximal inhibitory concentration (IC50 value) of widely used chemotherapy drugs for each patient. This package was constructed based on the chemical screening data from the Cancer Genome Project (CGP) dataset [15]. To explore drug response prediction by individual prognostic genes, we analyzed the pharmacological activity data of 218 FDA-approved drugs (Table S2) from the NCI-60 cancer cell lines that were downloaded from the CellMiner database Version2021.2 (https://discover.nci.nih.gov/cellminer).

### 2.8. Statistical Analysis

We identified DEGs among HCC and normal liver tissues using Wilcoxon signed-rank test. The K-M curve and the log-rank test were used to assess the difference in OS between groups. The correlation analysis was performed using the Pearson correlation coefficient. GraphPad Prism Version 7.0.4 and R software Version 4.0.2 were utilized to generate diagrams. *P* value < 0.05 was considered statistically significant.

## 3. Results

### 3.1. Construction of a Prognostic Gene Signature in the TCGA Cohort

**Figure 1:**
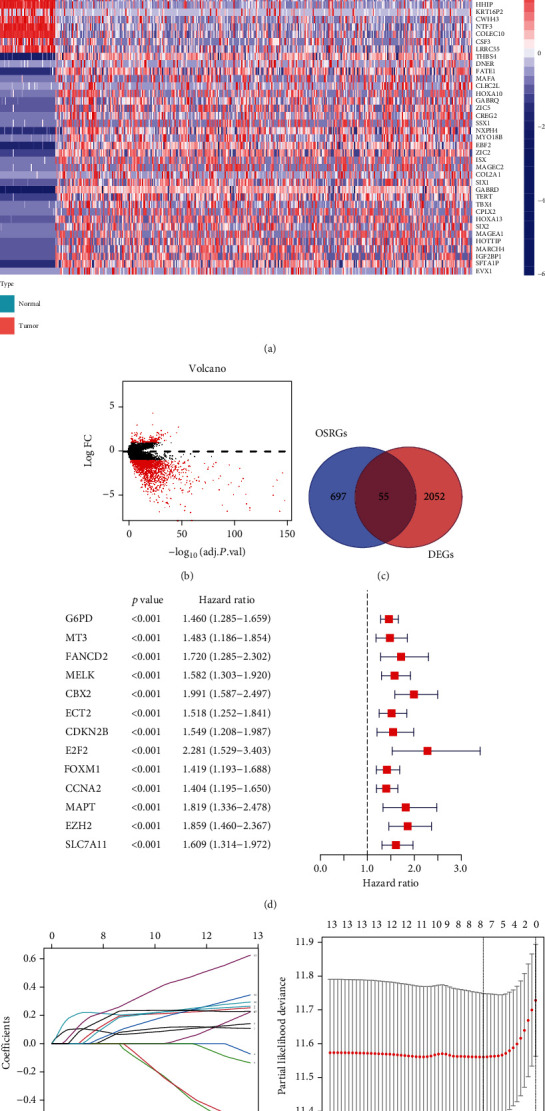
Construction of oxidative stress response genes (OSRGs) model. (a) Heatmap of the 30 up- and downregulated genes with the most significant differences. (b) Volcano plot of the differentially expressed genes (DEGs) in normal and HCC tissues. (c) Venn diagram of the shared genes of DEGs and OSRGs. (d) Screening of the candidate OSRGs using univariate Cox regression analysis. (e),(f) The LASSO Cox regression yielded a prognostic gene signature. Ten-fold cross-validation was used for the parameter chosen.

The TCGA-LIHC dataset contains 374 HCC patients, and the ICGC-LIRI-JP dataset contains 260 HCC patients. The clinical characteristics of these patients are displayed in Table 1. As illustrated in Figures 1(a) and 1(b), 2107 DEGs were identified in the TCGA cohort, among which 55 genes were OSRGs (Figure 1(c)). Thirteen OSRGs were verified to be significantly related to shorter OS (Figure 1(d)). After LASSO Cox regression analysis, an eight-gene prognostic signature was constructed (Figures 1(e) and 1(f)). To calculate the risk score of each patient, the following formula was used: risk score = 0.069∗expression level of *G*6*PD* + 0.177∗expression level of *MT*3 + 0.206∗expression level of *CBX*2 + 0.063∗expression level of *CDKN*2*B* + 0.078∗expression level of *CCNA*2 + 0.164∗expression level of *MAPT* + 0.248∗expression level of *EZH*2 + 0.213∗expression level of *SLC*7*A*11 (Figure S1). The above eight-gene expression was upregulated in HCC tissue compared with that in normal liver tissue in the TCGA cohort (Figure S2). We further validated the expression pattern of the proteins encoded by the above genes using clinical specimens in HPA database. All proteins except SLC7A11 were found to be elevated in HCC tissues. No conclusion could be drawn about SLC7A11 due to a lack of data. G6PD, MT3, and MAPT were exclusively located in the cytoplasm, CDKN2B was located in both the cytoplasm and nucleus, while CBX2, CCNA2, and EZH2 were located in the nucleus (Figure S3).

### 3.2. Validation of the Prognostic Gene Signature

**Figure 2:**
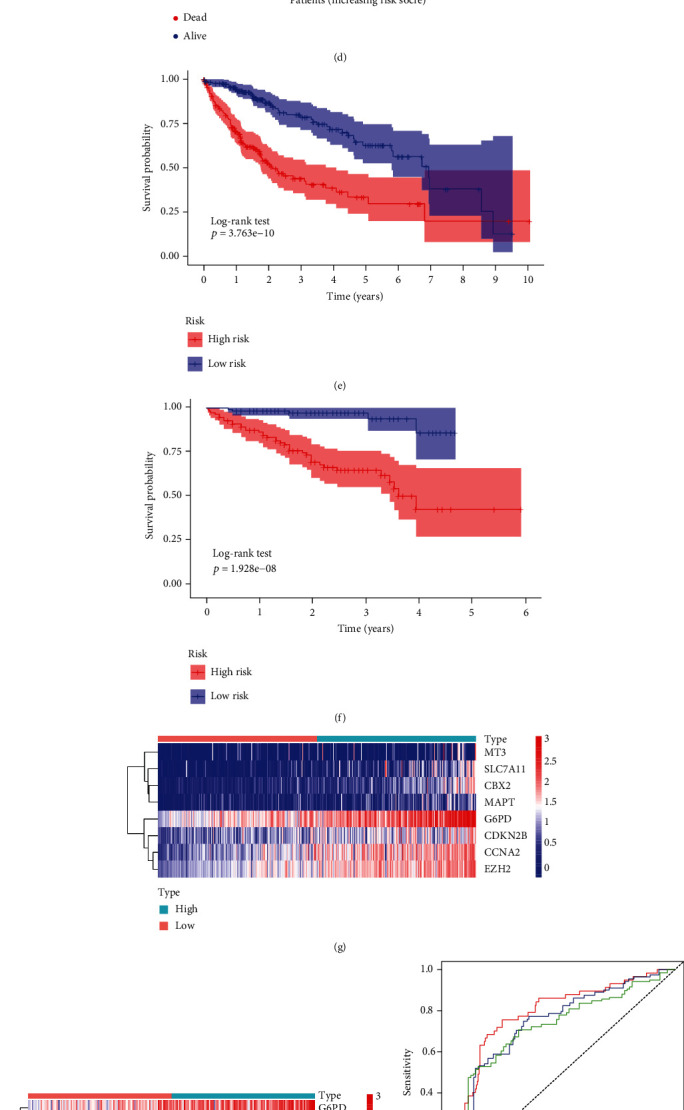
Prognostic performance of the gene signature. (a), (c), (e), (g), (i) TCGA cohort and (b), (d), (f), (h), (j) ICGC cohort. (a), (c) The distribution of patients with high- or low-risk scores. (b), (d) The survival status of each patient. (e), (f) Kaplan-Meier curve shows the different overall survival between the high- and low-risk groups. (g), (h) Heatmap shows the expression level of each gene in the gene signature. (i), (j) 1,2,3-year ROC curves and AUC for overall survival.

We next classified the patients into the high-risk score and low-risk score subgroups according to the median cut-off value of risk score in the TCGA and ICGC cohorts (Figure 2(a)). Patients in the high-risk group had a higher mortality rate than those did in the low-risk group (Figure 2(c)). In line with this, the K-M curve revealed a significantly shorter OS in the high-risk group (Figure 2(e)). The expression heatmap of the eight genes is displayed in Figure 2(g). Time-dependent ROC curves were used to assess the predictive accuracy of the prognostic gene signature. The area under the ROC curve (AUC) for the one-, two-, and three-year OS probabilities was 0.79, 0.75, and 0.73, respectively, which confirmed the effectiveness of this prognostic gene signature (Figure 2(i)). We then verified the robustness of the prognostic gene signature using the independent ICGC cohort. The results of patient distribution, K-M curve analysis, and AUC analysis were in line with those of the TCGA cohort, further supporting the robustness of the prognostic gene signature (Figures 2(b), 2(d), 2(f), 2(h), and 2(j)).

### 3.3. Evaluation of the Independent Prognostic Value of Risk Score and Nomogram Establishment

**Figure 3:**
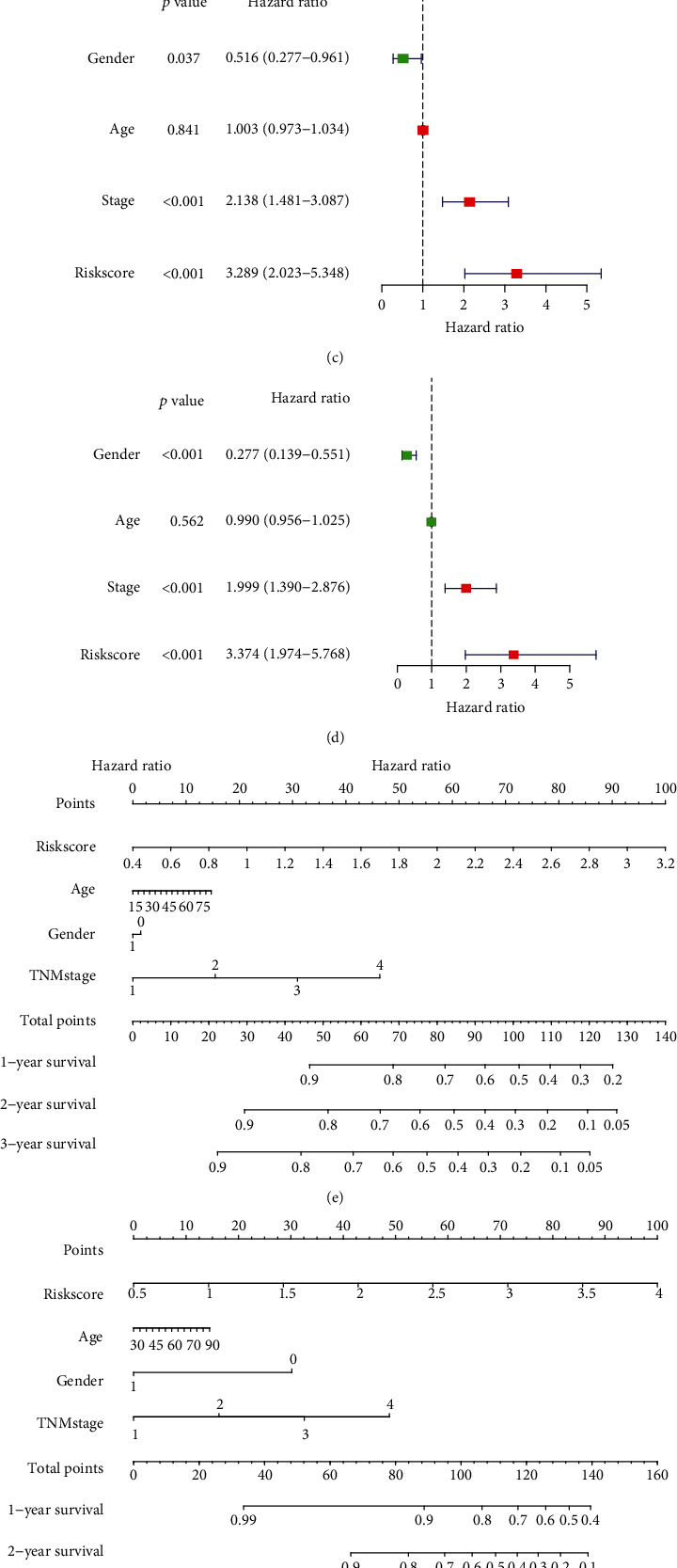
Identify the independent risk factors for OS and establishment of nomograms. (a), (b), (e), (g) TCGA cohort, (c), (d), (f), (h) ICGC cohort. (a), (c) Identify the OS-related risk factors using univariate Cox regression analysis. (b), (d) Multivariate Cox regression for assessing the independent risk factor for OS. (e), (f) Construction of nomograms with the risk scores and clinicopathological parameters. (g), (h) Calibration curves to evaluate the precision of nomograms.

We subjected the clinicopathological characteristics and risk scores into univariate and multivariate Cox regression analyses to identify independent prognostic predictors. Univariate Cox analysis of TCGA cohort revealed that stage, T, M, and risk score were significantly correlated with OS (Figure 3(a)). In the ICGC cohort, univariate Cox analysis suggested that female gender, stage, and risk score were strongly associated with OS (Figure 3(c)). After multivariate Cox analysis, risk score emerged as the only independent factor for efficiently predicting prognosis in both cohorts (Figures 3(b) and 3(d)). We then constructed nomograms combining clinical variables and risk scores to calculate the total score of each patient with HCC, which could predict the one-, two-, and three-year OS (Figures 3(e) and 3(f)). The calibration curves showed the excellent predictive accuracy of the nomograms (Figure 3(g) and 3(h)).

### 3.4. Immune Infiltration and ICI Therapy Prediction

**Figure 4:**
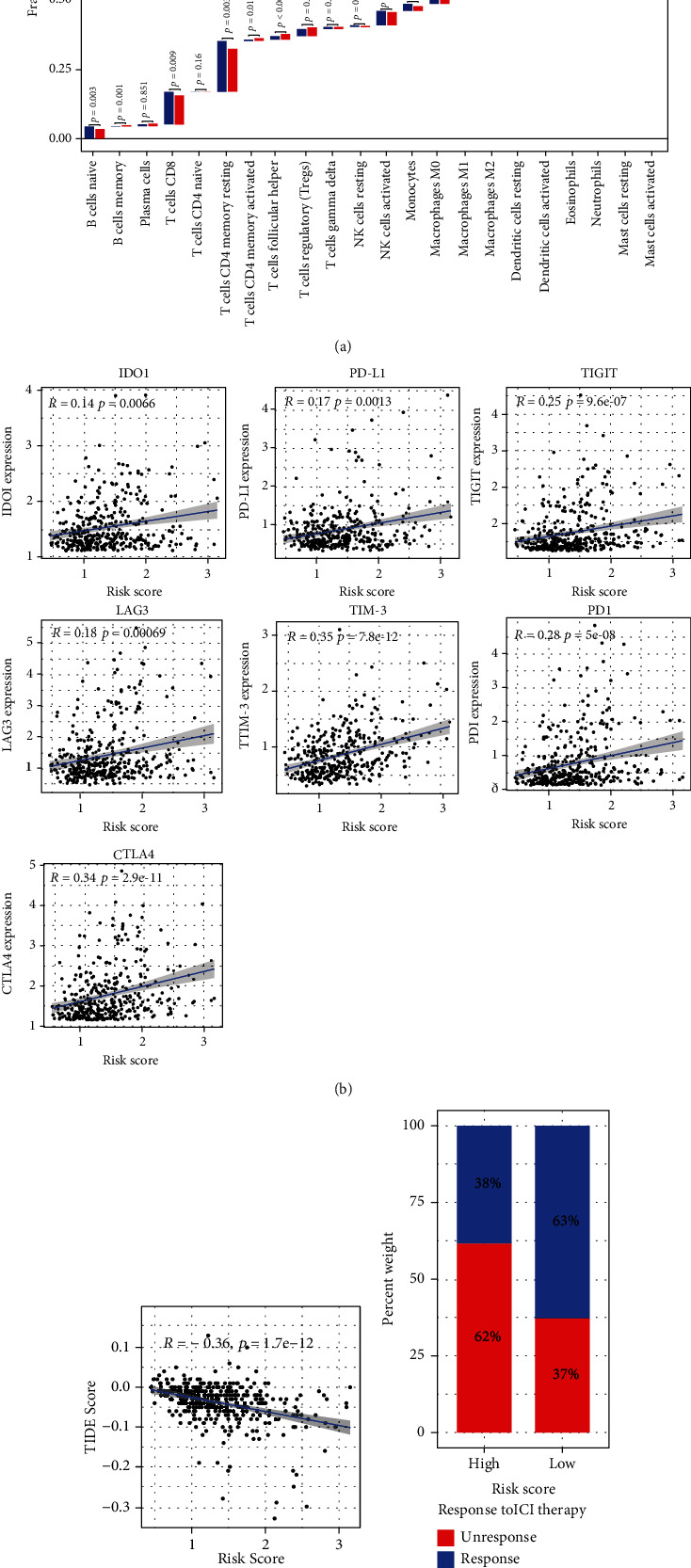
Association of the risk scores and the infiltration of immune cells and the response to ICI therapy. (a) The waterfall plot showed the fraction of infiltrating immune cells in high- and low-risk groups. (b) Association between the risk scores and expression level of indicated immune checkpoints. (c) Risk scores positively correlated to TIDE scores. (d) Responsive rate to ICI therapy in high- and low-risk groups. ICI: immune checkpoints inhibition.

Next, we employed the CIBERSORT algorithm to calculate the proportion of each type of infiltrated immune cell in individual HCC samples (Figure S4). As illustrated in Figure 4(a), the memory B cells, activated memory CD4 + T cells, follicular helper T cells, and M0 macrophages were more abundant in the high-risk group than the low-risk group. However, naïve B cells, CD8 + T cells, resting memory CD4+ T cells, monocytes, and eosinophils were reduced in the low-risk group. In addition, there was a positive correlation between the immune checkpoints expression level and risk scores (Figure 4(b)). TIDE analysis revealed that the risk scores, and TIDE scores were negatively correlated (Figure 4(c)). Consistently, the response rate to ICI therapy was expected to be higher in patients suffering from HCC with high risk scores (62% vs. 37%) (Figure 4(d)).

### 3.5. Associations of TMB with Risk Score in HCC Patients

**Figure 5:**
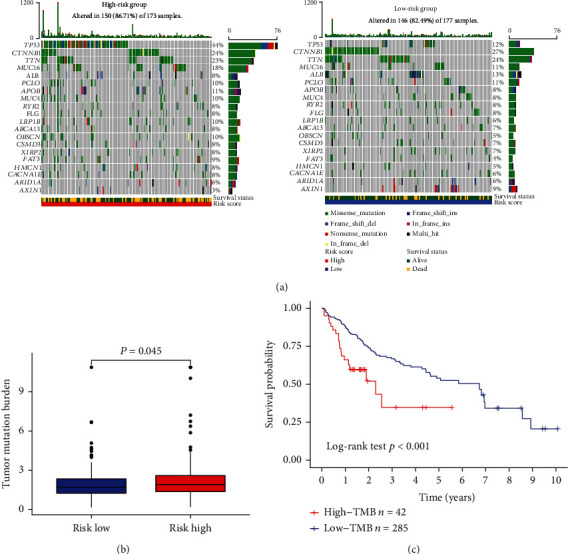
Tumor mutation burden (TMB) map. (a) The top 20 mutated genes in the high-risk and low-risk groups. (b) The tumor mutation burden scores were increased in the high-risk group. (c) Kaplan-Meier analysis shows the patients with high TMB scores have worse OS.

Further, we investigated the associations of TMB with the risk score. As mentioned before, we stratified HCC patients into high- or low-risk groups in the TCGA cohort. The comprehensive mutation data for each group were represented using a waterfall plot (Figure 5(a)). The five most frequently mutated genes were *TP53* (44%), *CTNNB1* (24%), *TTN* (23%), *MUC16* (18%), and *APOB* (11%) in the high-risk group, while *CTNNB1* (27%), *TTN* (23%), *ALB* (13%), *TP53* (12%), and *MUC16* (11%) were the top five in the low-risk group. In addition, the overall genome mutation occurrence rate was 86.71% and 82.49% in the high- and low-risk group, respectively. As displayed in Figure 5(b), the TMB scores were positively correlated with the risk scores. The K-M curve analysis revealed that the patients in the high-TMB group had shorter OS than those in the low-TMB group. (Figure 5(c)).

### 3.6. Analysis of the Correlation between Risk Score and Chemotherapy Sensitivity

**Figure 6:**
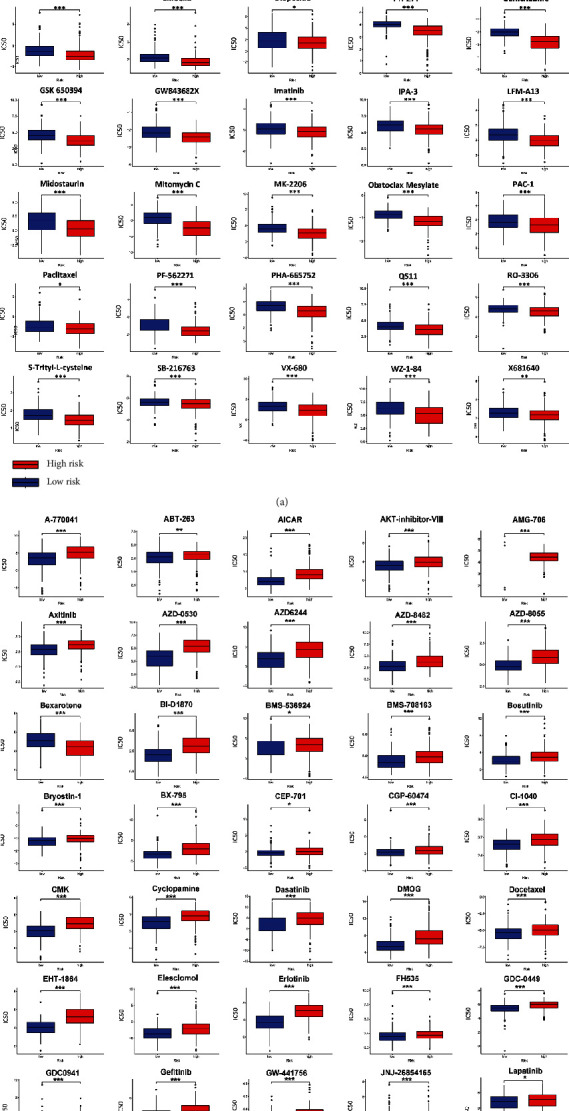
HCC patients with high risk scores were sensitive (a) or resistant (b) to indicated chemotherapy drugs (^∗^*P* < 0.05, ^∗∗^*P* < 0.01, and ^∗∗∗^*P* < 0.001).

**Figure 7:**
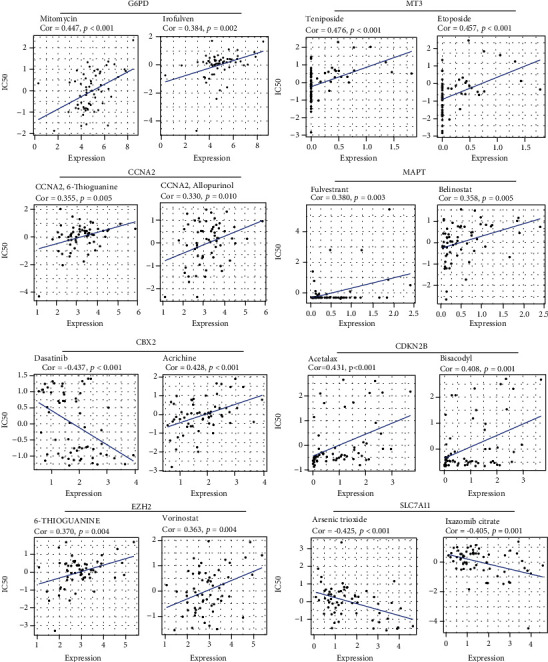
The correlation between the expression level of each prognostic gene and the drug response.

First, we evaluated the chemotherapeutic response using the drug activity and transcriptomic data from the CGP cell lines. In the high-risk group, HCC patients were sensitive to 45 chemotherapy and targeted therapy drugs and were resistant to other 44 drugs, as indicated by the variation of the IC50 (Figures 6(a) and 6(b)). For each prognostic gene in the gene signature, the corresponding expression level was analyzed in the transcriptomic data of NCI-60 cell lines. The top two FDA-approved drugs with the strongest positive or negative association with each gene are shown in Figure 7. The elevated expression of *G6PD*, *MT3*, *CBX2*, *CDKN2B*, *CCNA2*, *MAPT*, and *EZH2* was associated with increased resistance to mitomycin, teniposide, 6-thioguanine, and fulvestrant, etc. By contrast, elevated *CBX2* and *SLC7A11* expression is a hallmark of increased sensitivity to dasatinib, ixazomib citrate, and arsenic trioxide.

## 4. Discussion

Surgery is the first choice therapeutic strategy for HCC; however, most patients lose out on the opportunity to undergo tumor resection due to late diagnosis and large tumor sizes at the advanced stage. To overcome such challenges, in some studies, solutions, such as the use of novel synthetic materials to promote liver cell regeneration, have been proposed [16]. However, the bench-to-bedside translation of these technologies is still associated with considerable challenges. For unresectable HCC, selecting the appropriate strategy for comprehensive treatment, including chemotherapy, targeted therapy, and immunotherapy, is particularly vital for prolonging the survival of patients. Additionally, in this era of precision oncology, the conventionally used BCLC and AJCC staging systems do not enable the application of the precise prognostic and therapeutic recommendations for HCC. Therefore, exploring effective biomarkers for improving prognosis and providing excellent treatment strategies has become more important than ever. Therefore, in this study, for the first time, we developed a novel OSRG signature that could efficiently help clinicians predict HCC prognosis and choose a proper treatment strategy for individual patients.

In this study, we first identified DEGs between HCC tissues and normal liver tissues in the TCGA cohort. Then, the 55 genes shared by OSRGs and DEGs were analyzed using univariate Cox regression, which revealed that 13 of these genes were related to OS. An eight-gene signature was then established using the LASSO Cox regression analysis. The gene signature achieved excellent performance in predicting the OS rate in the TCGA and ICGC cohorts. In addition, the nomograms constructed with risk scores and clinicopathological factors showed good predictive ability for OS, and multivariate Cox analysis identified the risk score as an independent predictor of prognosis. The eight OSRGs contained in this signature are *G6PD*, *MT3*, *CBX2*, *CDKN2B*, *CCNA2*, *MAPT*, *EZH2*, and *SLC7A11*. Compared with normal tissue, these genes are highly expressed in HCC tissue and are associated with an unfavorable prognosis. *G6PD* encodes the protein glucose-6-phosphate dehydrogenase, which can resist damage by oxidative stress in cells [17]. Abnormal activation of G6PD contributes to the progression of many cancers [18]. *MT3* encodes the protein metallothionein3, which displays a solid ROS-scavenging capacity in oxidative stress-conditioned cells [19]. However, the exact role of MT3 in cancer cells remains controversial [20, 21]. As an oncogenic gene [22, 23], *CBX2* is strongly associated with genome-scale DNA methylation in various types of cancer cells, which is a response to micro-environmental stresses, particularly oxidative stress [24]. Recently, H-A Lee et al. reported that oxidative stress enhances *CDKN2B* expression and causes cell cycle arrest in HCC cells [25]. Nonetheless, the correlation between *CDKN2B* expression levels and prognosis is still unclear [26, 27]. *CCNA2* encodes Cyclin A2, which is a suppressor of intracellular oxidative stress [28] that is upregulated in colorectal and breast cancer tissues and is associated with poor prognosis [29, 30]. The *MAPT* encodes the protein Taus, which plays a significant role in the antioxidative response in neurons [31]. Elevated tau has been associated with a poor prognosis in prostate cancer [32]. *EZH2* is an oncogene that is upregulated under oxidative stress induced by H_2_O_2_ [33, 34]. Finally, SLC7A11 encodes a cystine/glutamate transporter that has been reported to suppress the expression of P-glycoprotein in breast cancer cells induced by ROS [35]. In addition, SLC7A11 was overexpressed in numerous cancers and is correlated with poor survival [36]. The above previous studies showed that all eight genes in our gene signature are related to oxidative stress response and most participate in the occurrence and development of tumors. These results also provide certain basic support for our study.

For unresectable HCC, immunotherapy plays a vital role in improving clinical outcomes. Unfortunately, current statistics show that response rates to immunotherapy remain low [37]. It is well recognized that the immune cells and immunosuppressive immune checkpoint molecules in the TME can greatly affect the effect of immunotherapy. However, the interaction between the tumor microenvironment and oxidative stress has barely been studied. In this study, we observed that the gene signature was closely associated with infiltrating immune cells in the tumor microenvironment as memory B cells, activated memory CD4 + T cells, follicular helper T cells, and M0 macrophages were abundant in patients with high risk scores. We also identified various immune checkpoints that positively correlate with risk scores, among which PD1, PD-L1, and CTLA4 are common targets for ICI therapy in clinical application. Preclinical studies showed that IDO1, TIGIT, LAG3, and TIM-3 are promising therapeutic targets [38]. Although patients with high expression levels of immune checkpoints tend to experience immune evasion and the consequent poor prognosis, the elevated expression of PD1, PD-L1, TIM-3, and IDO1 may result in a higher response rate to ICI therapy [39–41]. The results of TIDE analysis further supported these findings because patients with HCC having high risk scores were also associated with low TIDE scores, thereby indicating their high response rates to ICI treatment. Previous studies reported that high TMB indicates a better response to immunotherapy, which could be explained by the fact that mutant proteins encoded by mutated genes in cancer cells make tumors more immunogenic [42, 43]. This study found that the total gene mutation rate was higher in the high-risk group and that patients with high TMB scores had lower OS rates. Interestingly, the *TP53* mutation rate in the high-risk group was much higher than that in the low-risk group (44% vs. 27%). Some research findings suggested that the mutant p53 protein usually loses antioxidant function and increases intracellular ROS, which drives a function switch from a cancer suppressor protein to a cancer promoter protein [44]. Indeed, the existing research on how oxidative stress and OSRGs affect the immune components in the TME and the outcome of immunotherapy is still in the initial stage and worth further investigating.

Likewise, traditional cytotoxic chemotherapeutic drugs and targeted therapy drugs can still bring benefits for improving the prognosis of advanced HCC. In general, HCC cells are resistant to intravenous cytotoxic chemotherapy. Instead, transarterial chemoembolization and oral administration of sorafenib/lenvatinib are the globally accepted standards for treating advanced HCC. Doxorubicin and cisplatin are the frequently-used drugs in transarterial chemoembolization [45]. In the present study, the patients with HCC in the high-risk group exhibited elevated sensitivity to doxorubicin, afatinib, etoposide, and gemcitabine. Inversely, they were more likely to acquire resistance against sorafenib, the most extensively used multi-targeted tyrosine kinases inhibitor for treating late-stage HCC [46]. Using the NCI-60 data, we verified that each member of the gene signature could potentially indicate susceptibility to several FDA-approved chemotherapeutic drugs. Sensitivity towards some of these drugs has already been proved [30, 47–52]. For most of the prognostic genes, elevated expression levels are associated with increased drug resistance, making them potential targets to overcome this crisis. However, in HCC, the biological function of the OSRGs in drug response is not well studied; hence, more in-depth mechanistic research is worth exploring in a laboratory setting. For example, J. Sun et al. reported that cisplatin treatment induces ROS production in ovarian cancer and ROS promotes EZH2 expression, which inactivates AKT/ERK pathways that confers cisplatin resistance [53].

Presently, an increasing number of studies involving the use of multigene signatures for predicting the prognosis of HCC have been reported. In these studies, the researchers used m6A-related genes, energy metabolism-related genes, inflammation-related genes, immune response-related genes, and ferroptosis-related genes to construct their prognostic models [54–58]. Similar to this study, these five previous studies were conducted based on the expression data of the desired genes and involved the use of LASSO-COX regression to establish the optimal prognostic model. Further, in these studies, the AUC of the prognostic model for predicting 3-year OS was 0.65, 0.69, 0.61, 0.62, and 0.67, respectively, while in our study, the 3-year OS AUC reached 0.73. Given that AUC is an important indicator for measuring the sensitivity and specificity of diagnostic models, we believe that our gene signature showed better performance in terms of prognostic prediction. Moreover, we previously identified a 10-gene TME-related-gene signature as a prognostic classifier for HCC, with an AUC of 0.75 for 3-year OS [59]. However, in this present study, we achieved a similar performance using fewer genes, implying that this novel gene signature will be more attractive for clinical implementation. More importantly, the novelty of this study is not only in the fact that we describe a practical prognostic tool for HCC, but also that our results provide clinicians with a practical tool for selecting appropriate chemotherapy drugs. Due to the great heterogeneity that characterizes cancer cells, the sensitivity of HCC to various anticancer drugs varies widely. Compared with the findings reported in the abovementioned studies, our findings notably showed the existence of an association between the obtained OSRGs-based risk score and the IC50 of commonly used chemotherapy drugs. Similarly, predicting the response rate of HCC to ICI therapy is difficult considering the complicated component as well as the intricate interactions between different cell types in the TME. Some crucial factors that affect the efficacy of ICI therapy, such as the infiltrated immune cells, TMB, and the TIDE score, showed significant correlations with the obtained risk score. These novel findings offer promising prospects for enhancing antitumor effects by accurately providing patients with individualized treatment plans according to their different OSRGs-based risk scores.

There is no denying that some limitations exist in this study. Firstly, our gene signature was suited to the patients with surgical or biopsy specimens because this gene signature was based on gene expression data. Secondly, we used Cox regression analysis to screen the prognostic genes, which may lead to the overlook of some genes with biological significance. Thirdly, although we used a variety of methods to ensure the accuracy of our gene signature, the reliability of this model needs to be iteratively improved in long-term clinical applications with larger sample size.

## 5. Conclusions

By integrating the expression matrix of OSRGs as well as survival statistics based on two independent HCC cohorts, we established and validated a novel eight OSRGs-related gene signature for patients with HCC. This gene signature showed significant correlation between the TMB and immune cell infiltration in the TME. More importantly, its application could facilitate the estimation of the prognosis as well as therapeutic responses to immunotherapy and chemotherapy for patients with HCC. Notwithstanding, even though our gene signature showed good application prospects, it still needs further verification in clinical application to improve its stability. Taken together, our findings can help clinicians estimate the outcome of patients and select the appropriate therapeutic strategy for HCC to improve its prognosis. Our work also provides new insights into the diagnostic and therapeutic value of oxidative stress and OSRGs, laying a preliminary foundation for future mechanistic studies on OSRGs in HCC.

## Figures and Tables

**Table 1:** Clinicopathological characteristics of the patients with HCC.

Characteristics	TCGA		ICGC	
	Number of case	%	Number of case	%
Age				
≥60	204	54.1	210	80.8
<60	172	45.6	50	19.2
Unknown	1	0.3	0	0
Gender				
Male	255	67.6	192	73.8
Female	122	32.4	68	26.2
Grade				
1	55	14.6		
2	180	47.7		
3	124	32.9		
4	13	3.4		
Unknown	5	1.3	260	100
Stage				
1	175	46.4	40	15.4
2	87	23.1	117	45.0
3	86	22.8	80	30.8
4	5	1.3	23	8.8
Unknown	24	6.4	0	0
T				
1	185	49.1		
2	95	25.2		
3	81	21.5		
4	13	3.4		
Unknown	3	0.8	260	100
N				
0	257	68.2		
1	4	1.1		
Unknown	116	30.8	260	100
M				
0	272	72.1		
1	4	1.1		
Unknown	101	26.8	260	100

## Data Availability

The public datasets analyzed in this study can be downloaded here: https://portal.gdc.cancer.gov/repository and https://dcc.icgc.org/releases/current/Projects/LIRI-JP. All codes used in this study are available from the corresponding author upon reasonable request.

## Supplementary Materials

Supplementary Materials The supplementary material document contains all supplementary figures and tables cited in the main text.
