# Supplementary material for: Construction of a Novel Oxidative Stress Response-Related Gene Signature for Predicting the Prognosis and Therapeutic Responses in Hepatocellular Carcinoma
Source: Dis Markers. 2022 Sep 12;2022:6201987. doi: 10.1155/2022/6201987 (PMC9484914; doi:10.1155/2022/6201987)
Supplement: Supplementary Materials — The supplementary material document contains all supplementary figures and tables cited in the main text. [file 6201987.f1.zip › Supplemental figures.pdf]

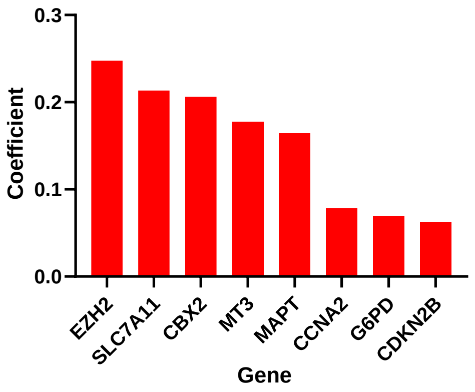

Figure. S1 Distribution of LASSO coefficients of each oxidative response genes.

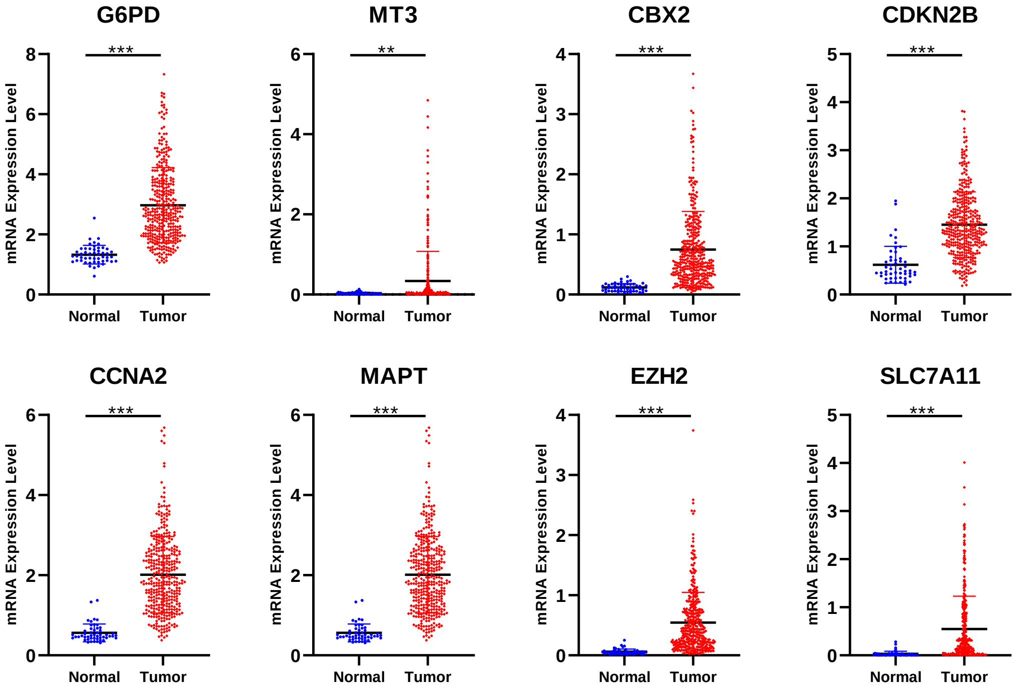

Figure. S2 The expression level of each gene of the gene signature in normal and HCC tissues. (**p<0.01, ***p<0.001)

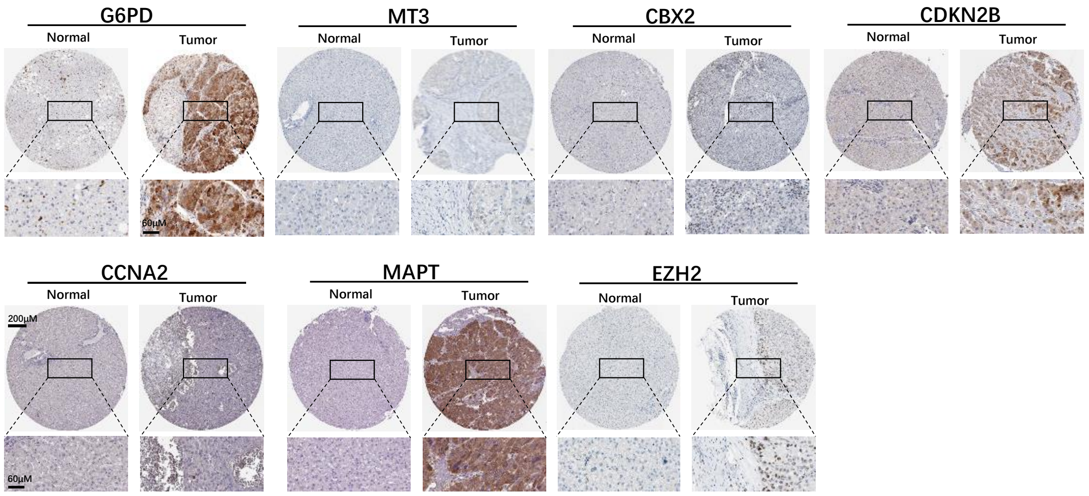

Figure. S3 Representative immunohistochemistry images of G6PD, MT3, CVX2, CDKN2B, CCNA2, MAPT, EZH2 in normal and HCC tissues.

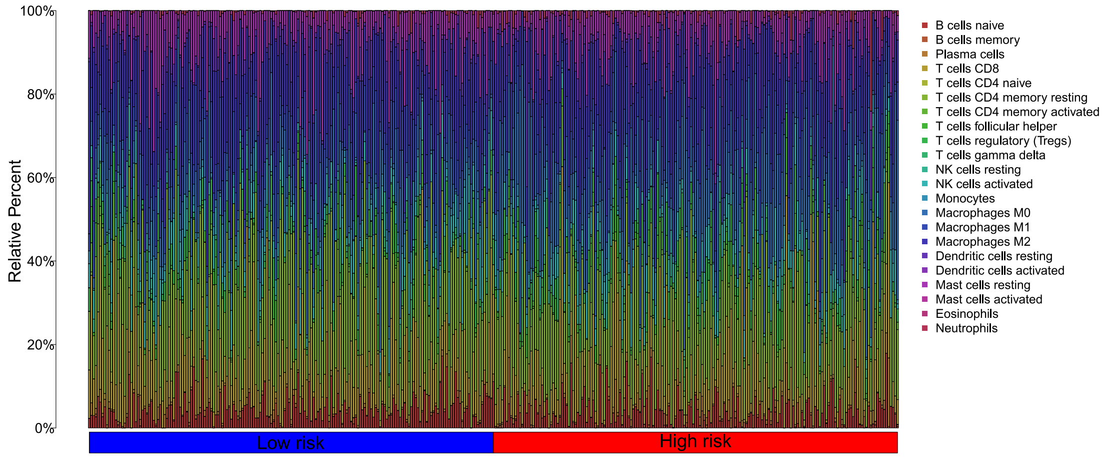

Figure. S4 The bar plot illustrated the proportion of each infiltrating immune cells of each HCC samples in low- or high risk groups in the TCGA cohort.
