# Supplementary material for: Construction of a Novel Oxidative Stress Response-Related Gene Signature for Predicting the Prognosis and Therapeutic Responses in Hepatocellular Carcinoma
Source: Dis Markers. 2022 Sep 12;2022:6201987. doi: 10.1155/2022/6201987 (PMC9484914; doi:10.1155/2022/6201987)
Supplement: Supplementary Materials — The supplementary material document contains all supplementary figures and tables cited in the main text. [file 6201987.f1.zip › Supplemental tables.docx]

**Table S1** Oxidative stress response–related genes.

| Gene symbol |
| --- |
| ABCC2 ABCD1 ABL1 ACOX2 ADA ADAM9 ADIPOQ ADNP2 ADPRS AGAP3 AIF1 AIFM2 AKR1C3 AKT1 ALAD ALDH3B1 ALOX5 ANGPTL7 ANKRD2 ANKZF1 ANXA1 APEX1 APOA4 APOD APOE APP APTX AQP1 ARG1 ARL6IP5 ARNT ARNTL ATF4 ATG7 ATOX1 ATP13A2 ATP2A2 ATP7A ATRN AXL BAD BAG5 BAK1 BCL2 BECN1 BMP7 BNIP3 BRF2 BTK C19orf12 CA3 CAMKK2 CAPN2 CASP3 CAT CBX8 CCL19 CCNA2 CCR7 CCS CD36 CD38 CDK2 CFLAR CHCHD2 CHD6 CHRNA4 CHUK CLN8 COA8 COL1A1 CPEB2 CRK CRYAB CRYGD CYBA CYBB CYCS CYGB CYP1B1 CYP2E1 DAPK1 DGKK DHCR24 DHFR DHFRP1 DHRS2 DIABLO DNM2 DPEP1 DUOX1 DUOX2 DUSP1 ECT2 EDN1 EEF2 EGFR EGLN1 EIF2S1 ENDOG EPAS1 EPX ERCC1 ERCC2 ERCC3 ERCC6 ERCC6L2 ERCC8 ERMP1 ERN1 ERO1A ETFDH ETS1 ETV5 EZH2 FABP1 FANCC FANCD2 FBLN5 FBXO7 FBXW7 FER FGF8 FKBP1B FOS FOSL1 FOXO1 FOXO3 FUT8 FXN FYN G6PD GATA4 GCH1 GCLC GCLM GGT7 GJB2 GLRX2 GNAO1 GPR37 GPR37L1 GPX1 GPX2 GPX3 GPX4 GPX5 GPX6 GPX7 GPX8 GSKIP GSR GSS GSTP1 GUCY1B1 H19 HAO1 HBA1 HBA2 HBB HDAC2 HDAC6 HGF HIF1A HMOX1 HMOX2 HNRNPD HNRNPM HP HSF1 HSPA1A HSPA1B HSPB1 HTRA2 HYAL1 HYAL2 IDH1 IL10 IL18RAP IL6 IMPACT INS IPCEF1 JAK2 JUN KCNA5 KCNC2 KDM6B KEAP1 KLF2 KLF4 KRT1 LANCL1 LDHA LIAS LONP1 LPO LRRK2 MACROH2A1 MAP1LC3A MAP3K5 MAPK1 MAPK13 MAPK3 MAPK7 MAPK8 MAPK9 MAPKAP1 MAPT MB MBL2 MCL1 MCTP1 MEAK7 MELK MET MGAT3 MGST1 MICB MIR103A1 MIR107 MIR132 MIR133A1 MIR17 MIR195 MIR19A MIR21 MIR29B1 MIR34A MIR675 MIR92A1 MIRLET7B MMP14 MMP2 MMP3 MMP9 MPO MPV17 MSRA MSRB2 MSRB3 MT-CO1 MT-ND1 MT-ND3 MT-ND5 MT-ND6 MT3 MTF1 MTR MYB MYEF2 NAPRT NCF1 NCF2 NCF4 NCOA7 NDUFA12 NDUFA6 NDUFB4 NDUFS2 NDUFS8 NEIL1 NET1 NFE2L1 NFE2L2 NME2 NME5 NME8 NOL3 NONO NOS3 NOX1 NOX4 NOX5 NQO1 NR4A2 NR4A3 NUDT1 NUDT15 NUDT2 OGG1 OSER1 OXR1 OXSR1 P4HB PAGE4 PARK7 PARP1 PAWR PAX2 PCGF2 PCNA PDCD10 PDE8A PDGFD PDGFRA PDGFRB PDK1 PDK2 PDLIM1 PENK PINK1 PJVK PKD2 PLA2R1 PLEKHA1 PLK3 PML PNKP PNPT1 PPARGC1A PPARGC1B PPIA PPIF PPP1R15B PPP2CB PPP5C PRDX1 PRDX2 PRDX3 PRDX4 PRDX5 PRDX6 PRKAA1 PRKAA2 PRKCD PRKD1 PRKN PRKRA PRNP PRODH PRR5L PSEN1 PSIP1 PSMB5 PTGS1 PTGS2 PTK2B PTPRK PTPRN PXDN PXDNL PXN PYCR1 PYCR2 PYROXD1 RACK1 RAD52 RBM11 RBPMS RELA REST RGS14 RHOB RIPK1 RIPK3 RNF112 ROMO1 RPS3 S100A7 SCARA3 SCGB1A1 SDC1 SELENOK SELENON SELENOP SELENOS SESN1 SESN2 SESN3 SETX SFPQ SGK2 SIGMAR1 SIN3A SIRPA SIRT1 SIRT2 SLC1A1 SLC23A2 SLC25A24 SLC7A11 SLC8A1 SMPD3 SNCA SOD1 SOD2 SOD3 SP1 SPHK1 SRC SRXN1 STAR STAU1 STK24 STK25 STK26 STOX1 STX2 STX4 SUMO4 TAT TBC1D24 THG1L TLDC2 TLR4 TLR6 TMEM161A TNFAIP3 TOR1A TP53 TP53INP1 TPM1 TPO TRA2B TRAF2 TRAP1 TREX1 TRPA1 TRPC6 TRPM2 TSC1 TXN TXN2 TXNIP TXNRD1 TXNRD2 UBE3A UBQLN1 UCN UCP1 UCP2 UCP3 VKORC1L1 VNN1 VRK2 WNT1 WNT16 WRN XRCC1 ZC3H12A ZNF277 ZNF580 ZNF622 AATF ABCD2 ACE2 ACOD1 ACOX1 ADGRB1 AGT AGTR1 AGTR2 AGXT2 ALOX12 ARF4 ARG2 ASS1 ATG5 ATP2B4 ATP5IF1 BCO2 BIRC2 BRCA1 BST1 CAV1 CCN1 CCN2 CCN6 CD177 CD34 CD47 CDKN1A CLCN3 CLEC7A CLU COQ7 CPS1 CRP CTNS CX3CR1 CYB5B CYB5R3 CYB5R4 CYP1A1 CYP1A2 DDAH1 DDAH2 DDIT4 DRD5 DUOXA1 DUOXA2 DYNLL1 EIF6 F2 F2RL1 FOXM1 FPR2 GADD45A GBF1 GCHFR GLA GLS2 GNAI2 GNAI3 GRB2 GRIN1 HBD HBE1 HBG1 HBG2 HBM HBQ1 HBZ HDAC4 HK2 HSP90AA1 HSP90AB1 HVCN1 ICAM1 IFI6 IFNG IL19 IL1B IMMP2L INAVA INSR ITGAM ITGB2 KHSRP LEP MAOB MAPK14 MIR181A2 MIR181B1 MIR199A1 MIR212 MIR24-1 MIR27B MIR590 MIR99B MMP8 MPV17L MT-CO2 MT-ND2 MTARC1 MTARC2 MTCO2P12 MTOR NCF1B NCF1C NDUFA13 NDUFS1 NDUFS3 NDUFS4 NNT NOS1 NOS1AP NOS2 NOX3 NOXA1 NOXO1 NQO2 NRROS P2RX4 PARL PDGFB PDK3 PDK4 PID1 PIKFYVE PLIN5 PMAIP1 PON3 PPARA PRCP PREX1 PRG3 PTGIS PTX3 RAB27A RAC1 RAC2 RFK RGN RNF41 ROCK2 RORA SFTPD SH3PXD2A SH3PXD2B SIRT3 SIRT5 SLC18A2 SLC25A33 SLC30A10 SLC5A3 SMAD3 SPHK2 SPR STK17A SYK TAFA4 TFAP2A TGFB1 TGFBR2 THBS1 TICAM1 TIGAR TLR2 TMEM106A TNF TSPO TUSC2 TYROBP VAV1 VDAC1 WDR35 XDH ZNF205 AKR1B1 CALU CTSD OAT PGAM1 ATP5F1B CAPRIN1 CLIC1 DCTN2 EIF5A HYOU1 VIM AGO1 AGO3 AGO4 BMI1 CBX2 CBX4 CBX6 CDK4 CDK6 CDKN2A CDKN2B CDKN2C CDKN2D E2F1 E2F2 E2F3 EED H2AB1 H2AC14 H2AC18 H2AC19 H2AC20 H2AC4 H2AC6 H2AC7 H2AC8 H2AJ H2AX H2AZ1 H2AZ2 H2BC1 H2BC10 H2BC11 H2BC12 H2BC13 H2BC14 H2BC15 H2BC17 H2BC21 H2BC3 H2BC4 H2BC5 H2BC6 H2BC7 H2BC8 H2BC9 H2BS1 H2BU1 H3-3A H3-3B H3C1 H3C10 H3C11 H3C12 H3C13 H3C14 H3C15 H3C2 H3C3 H3C4 H3C6 H3C7 H3C8 H4-16 H4C1 H4C11 H4C12 H4C13 H4C14 H4C15 H4C2 H4C3 H4C4 H4C5 H4C6 H4C8 H4C9 IFNB1 MAP2K3 MAP2K4 MAP2K6 MAP2K7 MAP4K4 MAPK10 MAPK11 MAPKAPK2 MAPKAPK3 MAPKAPK5 MDM2 MDM4 MINK1 MIR24-2 MOV10 PHC1 PHC2 PHC3 RBBP4 RBBP7 RING1 RNF2 RPS27A SCMH1 SUZ12 TFDP1 TFDP2 TNIK TNRC6A TNRC6B TNRC6C UBA52 UBB UBC GSTT2 JUNB MAOA MT1X NFIX NFKB1 SOD2-OT1 UGT1A6 |

**Table S2** FDA approved drugs in NCI-60 dataset.

| **Drug name** | **FDA status** | **PubChem SID** |
| --- | --- | --- |
| METHOTREXATE | FDA approved | 126941 |
| 6-THIOGUANINE | FDA approved | - |
| 6-MERCAPTOPURINE | FDA approved | - |
| Nitrogen mustard | FDA approved | - |
| Allopurinol | FDA approved | - |
| Actinomycin D | FDA approved | - |
| Chlorambucil | FDA approved | - |
| Thiotepa | FDA approved | - |
| Melphalan | FDA approved | - |
| Triethylenemelamine | FDA approved | 5799 |
| Dromostanolone Propionate | FDA approved | 224004 |
| Acrichine | FDA approved | - |
| Fluorouracil | FDA approved | - |
| Nandrolone phenpropionate | FDA approved | - |
| TESTOLACTONE | FDA approved | - |
| Mithramycin | FDA approved | - |
| Pipobroman | FDA approved | - |
| Cyclophosphamide | FDA approved | - |
| Mitomycin | FDA approved | 5746 |
| Floxuridine | FDA approved | - |
| Hydroxyurea | FDA approved | - |
| Uracil mustard | FDA approved | 6194 |
| Dexamethasone Decadron | FDA approved | - |
| Mitotane | FDA approved | - |
| DACARBAZINE | FDA approved | - |
| Vinblastine | FDA approved | - |
| Acetalax | FDA approved | - |
| Cytarabine | FDA approved | - |
| Vincristine | FDA approved | 249332 |
| Megestrol acetate | FDA approved | 11683 |
| tfdu | FDA approved | - |
| Procarbazine | FDA approved | 9703 |
| Lomustine | FDA approved | - |
| Daunorubicin | FDA approved | - |
| Daunorubicin | FDA approved | - |
| STREPTOZOCIN | FDA approved | - |
| Calusterone | FDA approved | 28204 |
| Estramustine | FDA approved | - |
| Vinblastine | FDA approved | - |
| Fluphenazine | FDA approved | 6215 |
| Arsenic trioxide | FDA approved | 261004 |
| AZACITIDINE | FDA approved | - |
| Cladribine | FDA approved | - |
| Mithramycin | FDA approved | - |
| Asparaginase | FDA approved | - |
| Ifosfamide | FDA approved | - |
| Acetalax | FDA approved | - |
| Fludarabine | FDA approved | - |
| Cisplatin | FDA approved | - |
| Isotretinoin | FDA approved | - |
| Teniposide | FDA approved | - |
| Doxorubicin | FDA approved | - |
| Fludarabine | FDA approved | - |
| Bleomycin | FDA approved | - |
| Paclitaxel | FDA approved | - |
| DECITABINE | FDA approved | - |
| Mitomycin | FDA approved | 281834 |
| Bendamustine | FDA approved | 77082 |
| Etoposide | FDA approved | - |
| Homoharringtonine | FDA approved | - |
| Mithramycin | FDA approved | - |
| Tegafur | FDA approved | - |
| Parthenolide | FDA approved | - |
| Dexrazoxane | FDA approved | - |
| Tamoxifen | FDA approved | - |
| PENTOSTATIN | FDA approved | - |
| RAPAMYCIN | FDA approved | - |
| Carboplatin | FDA approved | - |
| Valrubicin | FDA approved | - |
| Idarubicin | FDA approved | - |
| Epirubicin | FDA approved | - |
| Oxaliplatin | FDA approved | - |
| MITOXANTRONE | FDA approved | 4212 |
| Cytarabine | FDA approved | - |
| Mitoxantrone | FDA approved | - |
| Fludarabine | FDA approved | - |
| Imiquimod | FDA approved | - |
| Carmustine | FDA approved | - |
| Mithramycin | FDA approved | - |
| Rapamycin | FDA approved | - |
| Clofarabine | FDA approved | 354624 |
| Vinorelbine | FDA approved | 25136944 |
| Topotecan | FDA approved | - |
| Gemcitabine | FDA approved | 60750 |
| Bisacodyl, active ingredient of Viraplex | FDA approved | - |
| Irinotecan | FDA approved | - |
| Docetaxel | FDA approved | 148124 |
| Depsipeptide | FDA approved | - |
| Simvastatin | FDA approved | - |
| Raltitrexed | FDA approved | 135400182 |
| Midostaurin | FDA approved | 24202429 |
| 7-Ethyl-10-hydroxycamptothecin | FDA approved | 104842 |
| Bortezomib | FDA approved | - |
| Irofulven | FDA approved | 148189 |
| Temsirolimus | FDA approved | - |
| Denileukin Diftitox Ontak | FDA approved | - |
| Pemetrexed | FDA approved | 135410875 |
| Vorinostat | FDA approved | - |
| Estramustine | FDA approved | 54611422 |
| Arsenic trioxide | FDA approved | - |
| Eribulin mesilate | FDA approved | 54611489 |
| Gefitinib | FDA approved | - |
| Erlotinib | FDA approved | - |
| Fulvestrant | FDA approved | 104741 |
| Celecoxib | FDA approved | - |
| Zoledronate | FDA approved | 68740 |
| Belinostat | FDA approved | 6918638 |
| Lapatinib | FDA approved | 11679357 |
| Irinotecan | FDA approved | 60838 |
| Dasatinib | FDA approved | - |
| Everolimus | FDA approved | - |
| Pazopanib | FDA approved | 11525740 |
| Selumetinib | FDA approved | 10127622 |
| Imatinib | FDA approved | - |
| Lapatinib | FDA approved | 208908 |
| Nelfinavir | FDA approved | 64143 |
| Nilotinib | FDA approved | 644241 |
| Olaparib | FDA approved | 23725625 |
| Ixabepilone | FDA approved | 6445540 |
| Raloxifene | FDA approved | 5035 |
| Abiraterone | FDA approved | 132971 |
| Abiraterone | FDA approved | - |
| Sunitinib | FDA approved | 5329102 |
| Afatinib | FDA approved | 10184653 |
| Pazopanib | FDA approved | 10113978 |
| Olaparib | FDA approved | - |
| Depsipeptide | FDA approved | 5352062 |
| pralatrexate | FDA approved | 148121 |
| Pemetrexed | FDA approved | - |
| Bosutinib | FDA approved | - |
| Vismodegib | FDA approved | - |
| Actinomycin D | FDA approved | 45006140 |
| Mitomycin | FDA approved | 5702003 |
| Lenvatinib | FDA approved | - |
| Nelarabine | FDA approved | 3011155 |
| Crizotinib | FDA approved | 54613769 |
| DAUNORUBICIN | FDA approved | 30323 |
| DIGOXIN | FDA approved | 5702042 |
| ETHINYL ESTRADIOL | FDA approved | 24707 |
| Fluorouracil | FDA approved | - |
| Nitrogen mustard | FDA approved | 4033 |
| Melphalan | FDA approved | 460612 |
| 6-Thioguanine | FDA approved | - |
| TYROTHRICIN | FDA approved | 16132496 |
| Vinblastine | FDA approved | 9962911 |
| Cabozantinib | FDA approved | - |
| Neratinib | FDA approved | - |
| Axitinib | FDA approved | 6450551 |
| Etoposide | FDA approved | 6708815 |
| Azacitidine | FDA approved | - |
| Floxuridine | FDA approved | 5702211 |
| tepotinib | FDA approved | - |
| Trametinib | FDA approved | - |
| Palbociclib | FDA approved | - |
| Carfilzomib | FDA approved | - |
| Homoharringtonine | FDA approved | - |
| Ixazomib citrate | FDA approved | - |
| Teniposide | FDA approved | - |
| Ponatinib | FDA approved | - |
| Bleomycin | FDA approved | 60148416 |
| Paclitaxel | FDA approved | 441276 |
| Rapamycin | FDA approved | 60148419 |
| Teniposide | FDA approved | 6708778 |
| Simvastatin | FDA approved | - |
| Belinostat | FDA approved | - |
| Doxorubicin | FDA approved | 6916127 |
| Vincristine | FDA approved | 9832825 |
| Pipamperone | FDA approved | 4830 |
| Epirubicin | FDA approved | 65348 |
| Idelalisib | FDA approved | - |
| Topotecan | FDA approved | 60699 |
| ARSENIC TRIOXIDE | FDA approved | 518605 |
| 6-Mercaptopurine | FDA approved | - |
| Docetaxel | FDA approved | 9877265 |
| Vorinostat | FDA approved | - |
| Gefitinib | FDA approved | - |
| Clofarabine | FDA approved | 60148441 |
| Dasatinib | FDA approved | - |
| Irinotecan | FDA approved | - |
| VINORELBINE | FDA approved | 60780 |
| Copanlisib | FDA approved | - |
| Vandetanib | FDA approved | - |
| Cabozantinib | FDA approved | - |
| Panobinostat | FDA approved | - |
| brigatinib | FDA approved | - |
| Sonidegib | FDA approved | - |
| Sonidegib | FDA approved | - |
| Vemurafenib | FDA approved | 42611257 |
| Ibrutinib | FDA approved | - |
| Alectinib | FDA approved | - |
| ARRY-162 | FDA approved | - |
| Dabrafenib | FDA approved | - |
| Alectinib | FDA approved | - |
| BMN-673 | FDA approved | - |
| Bosutinib | FDA approved | - |
| Dacomitinib | FDA approved | - |
| ABT-199 | FDA approved | - |
| BMN-673 | FDA approved | - |
| Cobimetinib (isomer 1) | FDA approved | - |
| Cobimetinib (isomer 1) | FDA approved | - |
| LY-2835219 | FDA approved | - |
| IPI-145 | FDA approved | - |
| NMS-E628 | FDA approved | - |
| LDK-378 | FDA approved | - |
| LDK-378 | FDA approved | - |
| Encorafenib | FDA approved | - |
| Cobimetinib (isomer 1) | FDA approved | - |
| LEE-011 | FDA approved | - |
| Osimertinib | FDA approved | - |
| PF-06463922 | FDA approved | - |
| JNJ-42756493 | FDA approved | - |
| LOXO-101 | FDA approved | - |
| brigatinib | FDA approved | - |
| gilteritinib | FDA approved | - |
| Acalabrutinib | FDA approved | - |
| Sulfatinib | FDA approved | - |
| umbralisib | FDA approved | - |
| Copanlisib | FDA approved | - |
